# Modification by KCNE1 variants of the hERG potassium channel response to premature stimulation and to pharmacological inhibition

**DOI:** 10.1002/phy2.175

**Published:** 2013-11-29

**Authors:** Chunyun Du, Aziza El Harchi, Henggui Zhang, Jules C Hancox

**Affiliations:** 1 School of Physiology and Pharmacology and Cardiovascular Research Laboratories, Medical Sciences Building, University of BristolBristol, BS8 1TD, U.K.; 2 Biological Physics Group, School of Physics and Astronomy, University of ManchesterManchester, M13 9PL, U.K.

**Keywords:** Cardiac, cisapride, clarithromycin, hERG, KCNE1, Long QT, potassium channels, QT interval, quinidine

## Abstract

*human Ether-à-go-go-Related Gene* (*hERG*) encodes the pore-forming subunit of cardiac rapid delayed rectifier K^+^ current (*I*_Kr_) channels, which play important roles in ventricular repolarization, in protecting the myocardium from unwanted premature stimuli, and in drug-induced Long QT Syndrome (LQTS). KCNE1, a small transmembrane protein, can coassemble with hERG. However, it is not known how KCNE1 variants influence the channel's response to premature stimuli or if they influence the sensitivity of hERG to pharmacological inhibition. Accordingly, whole-cell patch-clamp measurements of hERG current (*I*_hERG_) were made at 37°C from hERG channels coexpressed with either wild-type (WT) KCNE1 or with one of three KCNE1 variants (A8V, D76N, and D85N). Under both conventional voltage clamp and ventricular action potential (AP) clamp, the amplitude of *I*_hERG_ was smaller for A8V, D76N, and D85N KCNE1 + hERG than for WT KCNE1 + hERG. Using paired AP commands, with the second AP waveform applied at varying time intervals following the first to mimic premature ventricular excitation, the response of *I*_hERG_ carried by each KCNE1 variant was reduced compared to that with WT KCNE1 + hERG. The *I*_hERG_ blocking potency of the antiarrhythmic drug quinidine was similar between WT KCNE1 and the three KCNE1 variants. However, the *I*_hERG_ inhibitory potency of the antibiotic clarithromycin and of the prokinetic drug cisapride was altered by KCNE1 variants. These results demonstrate that naturally occurring KCNE1 variants can reduce the response of hERG channels to premature excitation and also alter the sensitivity of hERG channels to inhibition by some drugs linked to acquired LQTS.

## Introduction

The rapid delayed rectifier potassium current (*I*_Kr_) contributes significantly to ventricular action potential (AP) repolarization and to setting the duration of the QT interval of the ECG (Tamargo et al. 2004; Sanguinetti and Tristani-Firouzi 2006). Due to distinct kinetic (rapid inactivation and reactivation/slow deactivation) properties, *I*_Kr_/hERG (*human Ether-à-go-go-Related Gene*) contributes to net membrane conductance for some time following complete AP repolarization, with the consequence that the channel can act to offset potentially arrhythmogenic premature depolarizations early in diastole (Lu et al. 2001; Vandenberg et al. 2001; McPate et al. 2009; Du et al. 2010). Under pathological situations such as acidosis, this protective role of *I*_Kr_/hERG is impaired (Du et al. 2010). Loss-of-function hERG mutations are well established to underlie the LQT2 form of congenital Long QT syndrome (LQTS; Vandenberg et al. 2001; Sanguinetti and Tristani-Firouzi 2006; Witchel and Hancox 2000) and, within the last decade, gain-of-function hERG mutations have been linked to the SQT1 form of the short QT syndrome (Brugada et al. 2004; Hong et al. 2005; Sun et al. 2011). *I*_Kr_/hERG channels are also recognized to comprise a major pharmacological target in the heart, both for antiarrhythmic (Class Ia and III) drugs and also for a wide range of noncardiac drugs linked to the acquired (drug-induced) form of the LQTS. Consequently, drugs in development are routinely tested for their ability to inhibit *I*_Kr_/hERG (Witchel and Hancox 2000; Vandenberg et al. 2001; Yap and Camm 2003; Hancox et al. 2008).

Although recombinant hERG channels expressed in mammalian cell lines pass ionic current (*I*_hERG_) at physiological temperature that is similar to *I*_Kr_ (Hancox et al. 1998; Zhou et al. 1998; Weerapura et al. 2002), it has been suggested that hERG may coassemble with an accessory protein from the KCNE family of single-transmembrane-domain proteins (for a review see Abbott et al. 2007). KCNE2 (also known as MiRP1) has received particular attention in this regard (e.g., Abbott et al. 1999; Larsen et al. 2001). Coexpression of the KCNE2 protein with hERG results in stable protein complexes and KCNE2 alters hERG channel unitary conductance, kinetics, and sensitivity to some antimicrobial agents (Abbott et al. 1999; Sesti et al. 2000). KCNE2 mutations are also implicated in the LQT6 form of LQTS (Lu et al. 2003; Kapplinger et al. 2009). However, KCNE2 can associate with multiple different ion channel partners both in vitro (Abbott et al. 2007) and in the myocardium (Jiang et al. 2004; Roepke et al. 2008) and experiments on canine hearts have revealed greater expression of KCNE2 in Purkinje fibres versus ventricular tissue (Pourrier et al. 2003), leading to the suggestion that KCNE2 may not coassemble with hERG outside of the cardiac conduction system (Sanguinetti and Tristani-Firouzi 2006; Hancox et al. 2008).

KCNE1 (also known as “minK”) is a 129 amino acid protein that is best known as the *β* subunit of channels responsible for the “slow” delayed rectifier K^+^ current, *I*_Ks_. Although it has been less well studied than KCNE2 in respect of influencing hERG, in the human heart KCNE1 levels are substantially greater than KCNE2 in both atrial and ventricular tissue (Bendahhou et al. 2005). McDonald and coworkers reported that coexpression of hERG with KCNE1 increased current density without a change in single-channel conductance, shifted activation gating leftward (by ∼6-10 mV) and that hERG-KCNE1 formed stable complexes (McDonald et al. 1997). Antisense oligonucleotide (AS-ODN) directed against KCNE1 has been reported to decrease *I*_Kr_ in both AT-1 cells and neonatal mouse ventricular myocytes (Yang et al. 1995; Ohyama et al. 2001). Additionally, in a study of K channel subunits in equine heart, KCNE1 was found to coimmunoprecipitate with equine ERG1 (Finley et al. 2002). Evidence that KCNE1 may modify *I*_Kr_ in humans comes from observations on LQTS-associated KCNE1 mutations. Bianchi et al. (1999) provided evidence that a LQT5 C-terminal KCNE1 mutation (D76N) could suppress *I*_hERG_ amplitude. In the same study, the KCNE1 V47F mutation was found to increase hERG current amplitude, but to a smaller extent than did wild-type (WT) KCNE1 (Bianchi et al. 1999). Other patient studies also provide evidence for clinically significant interactions between hERG and KCNE1. In one of these, a novel N-terminus KCNE1 mutation (A8V) was found in a female who exhibited palpitations, bradycardia, and marked QT interval prolongation (QT_c_ interval of 600 ms) (Ohno et al. 2007). Significantly, coexpression of A8V-KCNE1 with KCNQ1 produced little alteration in recombinant “*I*_Ks_,” but when the mutant was expressed with hERG it produced a marked suppression of *I*_hERG_ density (Ohno et al. 2007). The C-terminal D85N KCNE1 polymorphism has been reported to produce a dominant negative effect to reduce *I*_hERG_ (Nishio et al. 2009; Nof et al. 2011) and a recent report has highlighted a significantly higher incidence of D85N KCNE1 in patients with drug-induced LQTS than in population-based controls (Kaab et al. 2012).

Despite evidence that KCNE1 may influence *I*_Kr_/hERG in vivo and in vitro, at present there is no information as to whether or not KCNE1 variants can influence the protective role of *I*_Kr_/hERG against arrhythmogenic premature stimulation. Neither it is known whether KCNE1 variants exert a direct influence on the sensitivity of hERG to pharmacological blockade. This study was undertaken to address these issues, by comparing *I*_hERG_ elicited when hERG was expressed with three naturally occurring KCNE1 variants (A8V, D76N, and D85N) with that with WT KCNE1, using a combination of conventional voltage and AP voltage clamp measurements at physiological temperature.

## Material and Methods

### Constructs used and generation of KCNE1 variants

The common S38 KCNE1 polymorphism (in pCR3.1) was kindly donated by Dr. F. Toyoda (Shiga University of Medical Science, Japan). The more usual G38 KCNE1 WT variant was generated from this using QuickChange® site-directed mutagenesis (Stratagene, La Jolla, CA). This construct was then used as WT-KCNE1 and was also used as the template for construction of the A8V, D75N, and D85N KCNE1 mutations. For each variant, a pair of complementary oligonucleotide primers (shown in Table 1) was employed for mutation construction. All mutated cDNAs were sequenced to ensure that only the correct mutation had been made (Eurofins MWG Operon, Ebersberg, Germany).

**Table 1:** Primers for KCNE1 mutagenesis

S38G
Forward: 5′-ccccccgcagcggtgac-3′
Reverse: 5′-cttgccgtcaccgctgc-3′
A8V
Forward: 5′-ctgtctaacaccacagtggtgacgccctttctg-3′
Reverse: 5′-ctgtctaacaccacagcggtgacgccctttctg-3′
D76N
Forward: 5′-gctggagcactcgaacaacccattcaacgtcta-3′
Reverse: 5′-tagacgttgaatgggttgttcgagtgctccagc-3′
D85N
Forward: 5′-gctggagcactcgaacaacccattcaacgtcta-3′
Reverse: 5′-gctggagcactcgaacgacccattcaacgtcta-3′

### Cell culture and transfection

Human embryonic kidney (HEK 293) cells stably expressing hERG channels (generously donated by Dr. Craig January, University of Wisconsin) were maintained in Dulbecco's minimum essential medium (DMEM; Gibco, Life technologies, Paisley, U.K.) supplemented with 10% fetal bovine serum (Gibco, Life technologies), 50 *μ*g/mL gentamycin (Gibco, Life technologies) and 100 *μ*g/mL geneticin (G418; Gibco, Life technologies), and kept in a 5% CO_2_/95% O_2_ incubator at 37°C. Cells were passaged using enzyme free cell dissociation solution (Millipore, MA) and plated in 40-mm petri dishes containing DMEM medium supplemented with 10% fetal bovine serum and 50 *μ*g/mL gentamycin. Cells at >80% confluence were transiently transfected with 0.5 *μ*g of KCNE1 plasmids and 1 *μ*g of green fluorescent protein (GFP; in pCMX donated by Dr. Jeremy Tavare, University of Bristol, U.K.) using Lipofectamine 2000 (Invitrogen, Life technologies, Carlsbad, CA) according to the manufacturer's instructions. Cells were incubated at 37°C for at least 12 h prior to electrophysiological recording.

### Electrophysiological recording and solutions

Data acquisition and recording methods were identical to those employed in recent studies from our laboratory (Du et al. 2010, 2011a,b). Briefly, whole-cell voltage clamp recordings were made at 37°C with a superfusate containing (in mmol/L): 140 NaCl, 4 KCl, 2 CaCl_2_, 1 MgCl_2_, 10 Glucose, and 5 4-(2-hydroxyethyl)-1-piperazineethanesulfonic acid (HEPES) (titrated to pH of 7.45 with NaOH). The pipette solution contained (in mmol/L): 130 KCl, 1 MgCl_2_, 5 EGTA, 5 MgATP, and 10 HEPES (titrated to pH of 7.2 with KOH). Patch pipette resistance ranged from 2 to 4 MΩ. Series resistance values lay between 3 and 7 MΩ and were compensated by 60–80%. Data digitization rates were 10–25 kHz with an appropriate bandwidth of 2–10 kHz set on the amplifier. The ventricular AP waveforms for single and paired AP clamp experiments were identical to those used in previous investigations from our group (McPate et al. 2009; Du et al. 2010, 2011b).

Quinidine (gluconate), cisapride (monohydrate), and clarithromycin were obtained from Sigma-Aldrich (Dorset, U.K.) and were dissolved in deionized water (quinidine) and dimethyl sulfoxide (DMSO; cisapride and clarithromycin) to give an initial stock solution of 100 mmol/L, 10 mmol/L, and 50 mmol/L, respectively. Stock solutions were diluted appropriately and aliquots of drug stock solutions were added to Tyrode's solution to produce the final concentrations mentioned in the Results section. The final DMSO concentration in test solutions (for cisapride and clarithromycin) was 0.1% in experiments on WT KCNE1 and on the different KCNE1 variants. DMSO at concentrations up to 1% has been demonstrated previously not to affect significantly the potency of hERG blocking drugs (Du et al. 2006).

### Data analysis

Data were analyzed using Clampfit 10.0 (Molecular Devices, Sunnyvale, CA), Excel 2007, Origin 7, and Prism v3 (Graphpad Sofware Inc., La Jolla, CA) software. Data are presented as the mean ± standard error of the mean (SEM). For concentration–response relations, SEM values presented in the Results represent SEM values for the fit to the mean plotted data. Statistical comparisons were made using one-way or two-way analyses of variance (ANOVA) followed by Bonferroni's post hoc comparisons tests as appropriate. *P* values of less than 0.05 were considered to be statistically significant.

Mathematical fits to the data were as follows: The voltage dependence of *I*_hERG_ activation was fitted with a Boltzmann equation of the form:


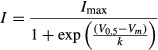
(1)

where *I* is the peak *I*_hERG_ tail current amplitude at the test voltage *V*_*m*_; *I*_max_ is the maximal tail current observed; *V*_0.5_ is the voltage at which *I*_hERG_ was half-maximally activated; and *k* is the slope factor for the fitted relationship.

The voltage dependence of *I*_hERG_ inactivation was fitted with a modified Boltzmann equation:


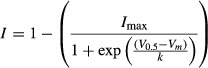
(2)

where *V*_0.5_ is the half-maximal inactivation voltage and other terms have same meaning as for equation 1.

The time course of *I*_hERG_ recovery from inactivation was fitted with a one-phase exponential association:


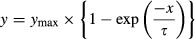
(3)

where *y* is the current amplitude at time *x*; *y*_max_ is the maximal *I*_hERG_ transient observed; and *τ* presents the time constant of *I*_hERG_ recovery from inactivation.

The fractional block of hERG tail current by drugs was determined using an equation of the form:


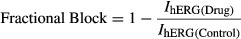
(4)

where *I*_hERG(Control)_ is the amplitude of *I*_tail_ in control and *I*_hERG(Drug)_ is the *I*_tail_ amplitude in the presence of the drug.

The relationship between drug concentration and *I*_hERG_ fractional block was determined by fitting data with a Hill equation of the form:


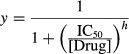
(5)

where *y* is fractional block at a given concentration (on a logarithmic scale) of Drug ([Drug]), IC_50_ is the Drug concentration producing half-maximal inhibition of *I*_hERG_, and *h* is the Hill coefficient for the fit to the plotted data. No more than two drug concentrations were studied in a single experiment and consequently fits with equation 5 were made to the mean pooled fractional block data.

## Results

### KCNE1 variants alter the amplitude of *I*_hERG_ elicited under AP clamp and conventional voltage clamp

**Figure 1:**
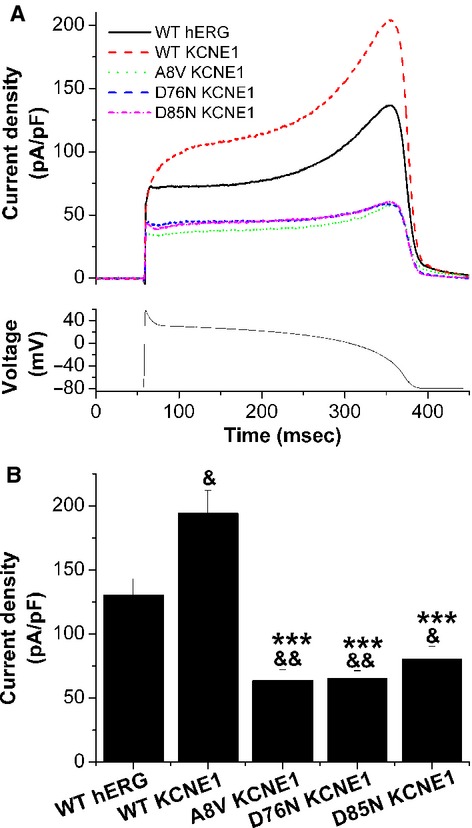
Effect of KCNE1 variants on *I*_hERG_ elicited by action potential clamp (AP clamp). (A) Representative *I*_hERG_ for hERG alone, wild-type (WT)-KCNE1 + hERG, A8V-KCNE1 + hERG, D76N-KCNE1 + hERG, and D85N-KCNE1 + hERG, elicited by the ventricular AP voltage command shown as the lower trace. (B) Bar charts showing the mean (±SEM) peak *I*_hERG_ elicited during the repolarization phase of the applied AP command; data were normalized to cell capacitance. *n* = 6 cells for WT hERG; *n* = 5 cells for WT-KCNE1 hERG; *n* = 5 cells for A8V-KCNE1 hERG; *n* = 6 cells for D76N-KCNE1 hERG, and *n* = 7 cells for D85N-KCNE1 hERG. ^&^*P* < 0.05 compared with WT hERG; ^&&^*P* < 0.01 compared with WT hERG; ***P < 0.001 compared with hERG+WT KCNE1. hERG, human Ether-à-go-go-Related Gene.

The effect of WT KCNE1 and KCNE1 variants on *I*_hERG_ amplitude under AP clamp (Hancox et al. 1998) was first studied. A ventricular AP waveform generated by the ten Tusscher et al. (2004) human ventricle model, employed in prior studies from our laboratory (Du et al. 2010, 2011b), was used as the voltage command (Fig. 1A, lower panel). Example *I*_hERG_ traces during the AP command waveform are shown in Figure 1A (upper panel), normalized to cell membrane capacitance in order to facilitate comparison. Consistent with prior observations under conventional voltage clamp (McDonald et al. 1997), the magnitude of *I*_hERG_ with WT KCNE1 was greater than that for hERG alone, whereas for each of the KCNE1 variants (A8V, D76N, and D85N) studied, the response was smaller than that for WT KCNE1 or hERG alone. Mean data for peak *I*_hERG_ density during repolarization are shown in Figure 1B, demonstrating significant suppression of *I*_hERG_ magnitude by each KCNE1 mutation compared to WT KCNE1 or hERG alone.

**Figure 2:**
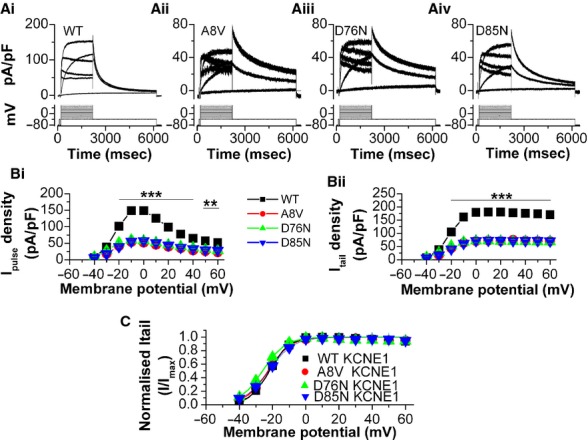
Current–voltage (*I*–*V*) relationships for *I*_hERG_ with different KCNE1 variants. (A) Example *I*_hERG_ records elicited by the voltage protocol shown in the lower panel for the different KCNE1 variants examined. (A) Wild-type (WT) KCNE1; (ii) A8V KCNE1; (iii) D76N KCNE1; (iv) D85N KCNE1. For clarity of display currents at selected test voltages (−40, −20, 0, 20, 40, and 60 mV) are shown. Note that the *y*-axis scale in (ii–iv) differs from that in (i). (B) *I*–*V* relations for end-pulse current (i) and tail current at −40 mV (ii) carried by WT-KCNE1 + hERG and by hERG with each of the three KCNE1 mutant variants. Data were normalized to the maximal current amplitude observed with WT-KCNE1 + hERG and cell capacitance. For (i), **P < 0.01 for A8V KCNE1, D76N KCNE1, and D85N KCNE1 compared with WT KCNE1 except for D76N KCNE1 and D85N KCNE1 at 60 mV where *P* < 0.05; For both (i and ii) ***P < 0.001 for A8V KCNE1, D76N KCNE1, and D85N KCNE1 compared with WT KCNE1 except for D76N KCNE1 at 40 mV where *P* < 0.01. Note that error bars for some of the points are small and are obscured by the symbols. (C). The steady-state activation plots derived from *I*_tail_ measurements. For each cell and each expression condition, *I*_tail_ measurements were normalized to the maximal *I*_tail_ during the protocol. The *I*–*V* relations were fitted by equation 1 (Material and Methods). There was no significant difference for the *V*_0.5_ and *k* values among the four groups. Note that error bars for some of the points are small and are obscured by the symbols. For B and C, *n* = 7 cells for WT-KCNE1 + hERG; *n* = 7 cells for A8V-KCNE1 + hERG; *n* = 6 cells for D76N-KCNE1 + hERG; and *n* = 7 cells for D85N-KCNE1 + hERG. hERG, human Ether-à-go-go-Related Gene.

Figure 2A shows families of *I*_hERG_ currents (upper traces; normalized to membrane capacitance) for *I*_hERG_ with WT KCNE1 and the three variants studied, elicited by conventional voltage clamp (protocol shown in lower traces). Similar to the data with AP clamp, current magnitude was suppressed by the A8V, D76N, and D85N KCNE1 variants. Figure 2B shows mean current–voltage (*I*–*V*) relations for end pulse current (Fig. 2B-i) and tail current (*I*_tail_; Fig. 2B-ii), demonstrating marked *I*_hERG_ suppression in the presence of KCNE1 mutations over the majority of the voltage range studied. Figure 2C shows normalized *I*_tail_-voltage plots with Boltzmann fits to the data to obtain activation *V*_0.5_ and *k* values. The derived *V*_0.5_ values were −21.89 ± 1.09 mV (*k* = 5.64 ± 0.12, *n* = 7 cells) for WT KCNE1-hERG, −22.64 ± 1.13 mV (*k* = 6.46 ± 0.15, *n* = 7 cells) for A8V KCNE1-hERG, −25.94 ± 1.63 mV (*k* = 6.19 ± 0.26, *n* = 6 cells) for D76N KCNE1-hERG, and −22.33 ± 1.99 mV (*k* = 6.80 ± 0.22, *n* = 7 cells) for D85N KCNE1-hERG. There was no significant difference for the *V*_0.5_ and *k* values among these four groups. However, these values exhibited a modest negative shift compared to that for WT *I*_hERG_ alone (*V*_0.5_ of −16.06 ± 0.97 mV and *k* of 6.23 ± 0.23). Our data for hERG expressed alone compared to that with hERG + WT KCNE1 coexpression in respect of current amplitude and activation *V*_0.5_ shift are consistent with prior findings under conventional voltage clamp (McDonald et al. 1997). In consequence, subsequent experiments focused on comparisons between WT KCNE1 and variant KCNE1 expression conditions.

### Effect of KCNE1 variants on *I*_hERG_ inactivation properties

**Figure 3:**
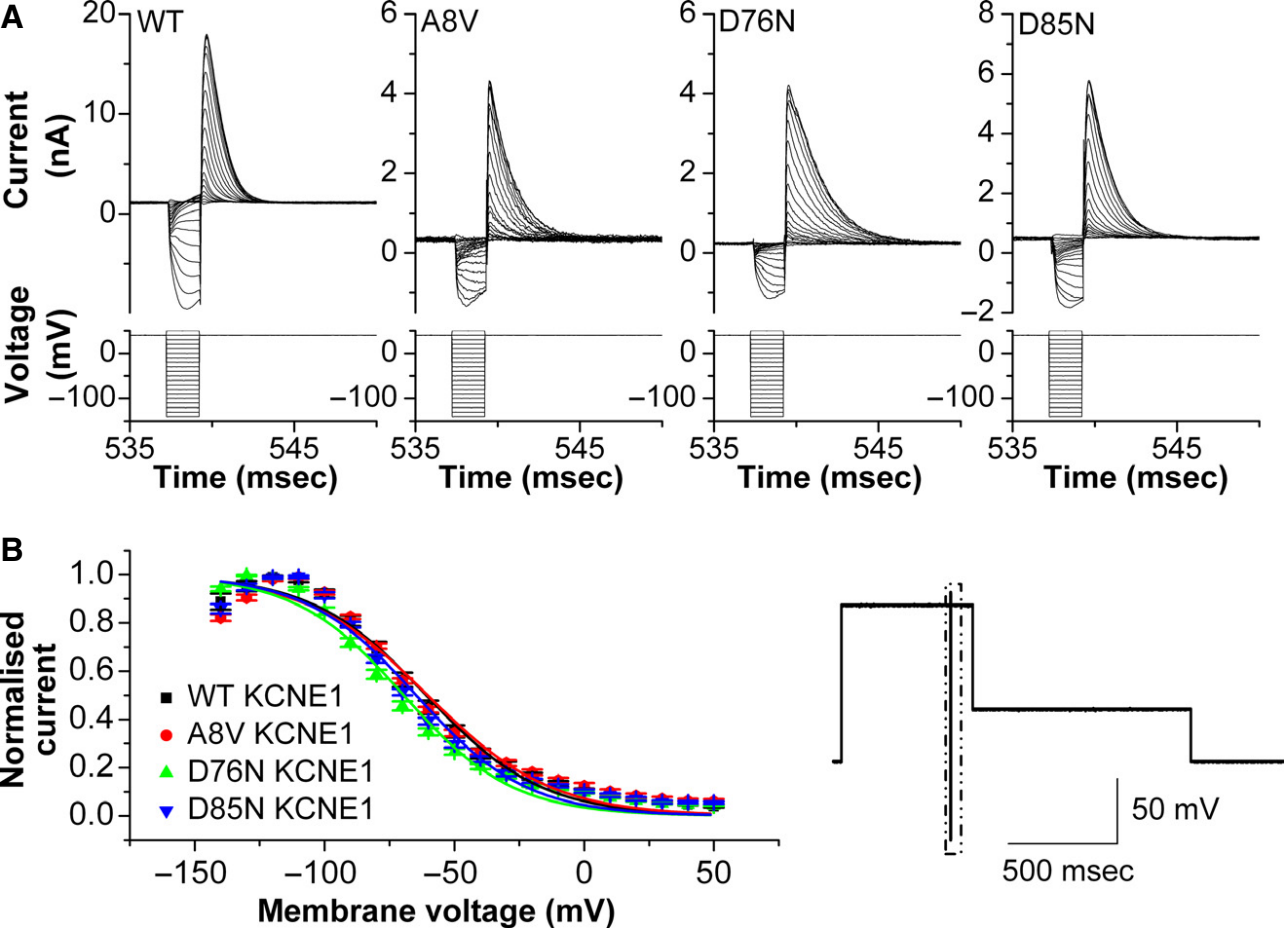
Voltage dependence of *I*_hERG_ inactivation with different KCNE1 variants. (A) Representative families of *I*_hERG_ on an enlarged scale elicited by the protocol shown as an inset to panel B. Note that *y*-axis scale differs between the different KCNE1 variants. (B) Plots against voltage (during the second step of the protocol) of *I*_hERG_ availability for the wild-type (WT)-KCNE1 hERG and three other KCNE1 variants. The availability plot was constructed as described in the Results and the data were then fitted with equation 2. *n* = 6 cells for WT-KCNE1 + hERG; *n* = 7 cells for A8V-KCNE1 + hERG; *n* = 7 cells for D76N-KCNE1 + hERG; and *n* = 8 cells for D85N-KCNE1 + hERG. The inset to panel B shows the voltage protocol used; the boxed area highlights the “ladder” of brief repolarizing steps shown at faster time-base in panel A. *V*_0.5_ and *k* values are given in the Results text. Inactivation *V*_0.5_ did not differ significantly between the variants except for D76N KCNE1-hERG (*V*_0.5_ *P* < 0.01 vs. WT and vs. A8V KCNE1). Note that error bars for some of the points are small and are obscured by the symbols. hERG, human Ether-à-go-go-Related Gene.

Figure 3A (upper panels) shows families of *I*_hERG_ elicited by the protocol shown in the lower panels (and inset to Fig. 3B; [Zou et al. 1998; McPate et al. 2005; Du et al. 2010]). The protocol was comprised of three principal voltage steps: an initial depolarizing step to activate and inactivate *I*_hERG_; a second brief repolarizing step to induce recovery from inactivation at different voltages; and a third depolarizing step that elicited an *I*_hERG_ transient, the magnitude of which depended on the availability of *I*_hERG_ subsequent to the brief repolarization step (Zou et al. 1998; McPate et al. 2005; Du et al. 2010). Following this, the membrane potential was progressively returned to the holding potential (with a step first to −40 mV and then return to −80 mV). Currents elicited by the first three steps were analyzed. Figure 3A displays the ladder of brief repolarizing steps (step 2) and the *I*_hERG_ transients elicited by the third step. WT KCNE1 and the three mutant variants were tested. The current elicited during the third step, following each repolarization voltage, was normalized to the maximal current amplitude during the protocol and mean (±SEM) data were then plotted as shown in Figure 3B. Data were corrected for residual capacitative transients by fitting the current during the third (depolarization) step with a single-exponential function and extrapolating back to the start of the third step (Zou et al. 1998; McPate et al. 2005; Du et al. 2010). The resulting plots were fitted with a modified Boltzmann equation to obtain an inactivation *V*_0.5_ of −60.75 ± 2.19 mV (*k* = 21.88 ± 0.82, *n* = 6 cells) for WT KCNE1-hERG, −60.06 ± 1.21 mV (*k* = 23.73 ± 1.2, *n* = 7 cells) for A8V KCNE1-hERG (*V*_0.5_ *P* > 0.05 vs. WT), −69.53 ± 1.48 mV (*k* = 20.74 ± 0.31, *n* = 7 cells) for D76N KCNE1-hERG (*V*_0.5_ *P* < 0.01 vs. WT and vs. A8V), and −64.93 ±1.24 mV (*k* = 20.62 ± 0.74, *n* = 8 cells) for D85N KCNE1-hERG (*V*_0.5_ *P* > 0.05 vs. WT). Thus, the inactivation curve was negatively shifted for D76N KCNE1 compared with WT KCNE1 and with A8V KCNE1. The time course of development of inactivation was compared between WT KCNE1 and the three other variants by monoexponential fitting of the decline of the *I*_hERG_ elicited by the third step following a repolarization voltage of −120 mV. The time constant of *I*_hERG_ inactivation at −120 mV was 1.15 ± 0.12 msec for WT KCNE1-hERG, 1.29 ± 0.09 msec for A8V KCNE1-hERG, 1.64 ± 0.16 msec for D76N KCNE1-hERG, and 1.19 ± 0.10 for D85N KCNE1-hERG. Though inactivation of D76N KCNE1-hERG appeared to develop slightly slower than that for the other variants, there was no significant difference in the time constant among the four variants (*P* > 0.05).

**Figure 4:**
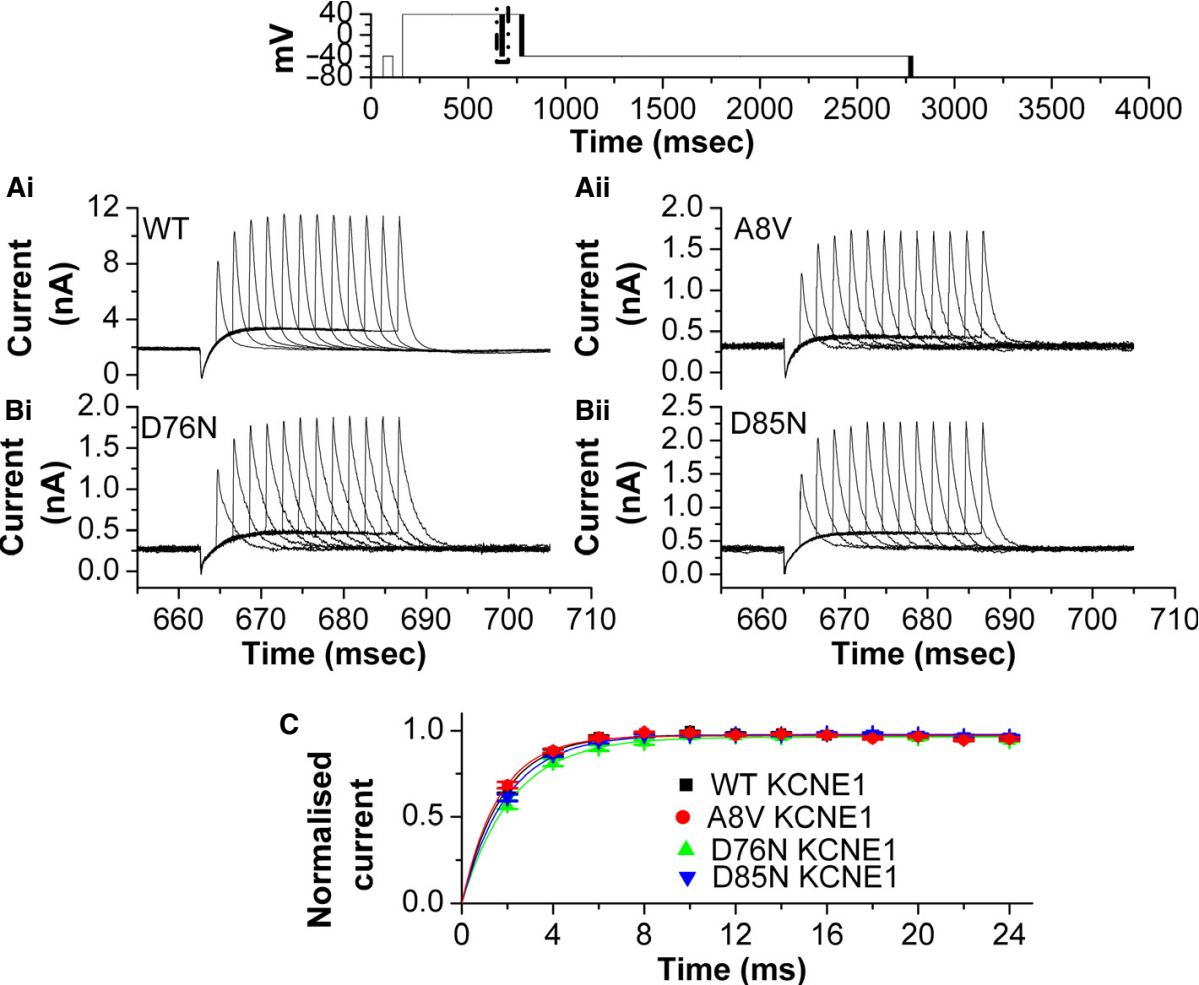
Recovery of *I*_hERG_ from inactivation with different KCNE1 variants. (A) *I*_hERG_ transient records (during the boxed area of the protocol shown as an inset) for wild-type (WT)- KCNE1 + hERG (i) and A8V-KCNE1 + hERG (ii). (B) *I*_hERG_ transient records for D76N-KCNE1 + hERG (i) and D85N-KCNE1 + hERG (ii) elicited by the same protocol as the data in panel A. (C) Plots of *I*_hERG_ recovery transient amplitude against time. The peak current amplitude upon the second step to +40 mV following different duration repolarization steps was normalized to the maximum current observed during the +40 mV step The relationship was fitted with equation 3. *n* = 6 cells for WT-KCNE1 + hERG; *n* = 7 cells for A8V-KCNE1 + hERG; *n* = 8 cells for D76N-KCNE1 + hERG; and *n* = 7 cells for D85N-KCNE1 + hERG. Time constant values are given in the Results text. That for D76N differed from the value for A8V (*P* < 0.05) but not from WT KCNE1. Note that error bars for some of the points are small and are obscured by the symbols. hERG, human Ether-à-go-go-Related Gene.

The time course of recovery of *I*_hERG_ from inactivation was evaluated with the protocol shown in the inset of Figure 4A (McPate et al. 2009; Du et al. 2010). *I*_hERG_ transient records for WT KCNE1 and A8V KCNE1 are shown in Figure 4A-i and A-ii, whereas those for D76N and D85N are shown in Figure 4B-i and B-ii. The outward transient current representing *I*_hERG_ recovery following repolarizing steps of different durations was measured and normalized to the maximal transient current observed during the protocol and was plotted against the corresponding duration of the repolarization step (McPate et al. 2009; Du et al. 2010) (Fig. 4C). The resulting data were fitted with a monoexponential association to give time constant values of 1.77 ± 0.08 msec (*n* = 6 cells) for WT KCNE1-hERG, 1.68 ± 0.09 msec (*n* = 7 cells) for A8V KCNE1-hERG, 2.23 ± 0.16 msec (*n* = 8 cells) for D76N KCNE1-hERG, and 2.03 ± 0.08 mV (*n* = 7 cells) for D85N KCNE1-hERG. The recovery from inactivation of *I*_hERG_ with D76N KCNE1 was slower than that with A8V KCNE1 (*P* < 0.05), but not than that with WT KCNE1.

### Effect of KCNE1 variants on the response of *I*_hERG_ to premature stimuli

**Figure 5:**
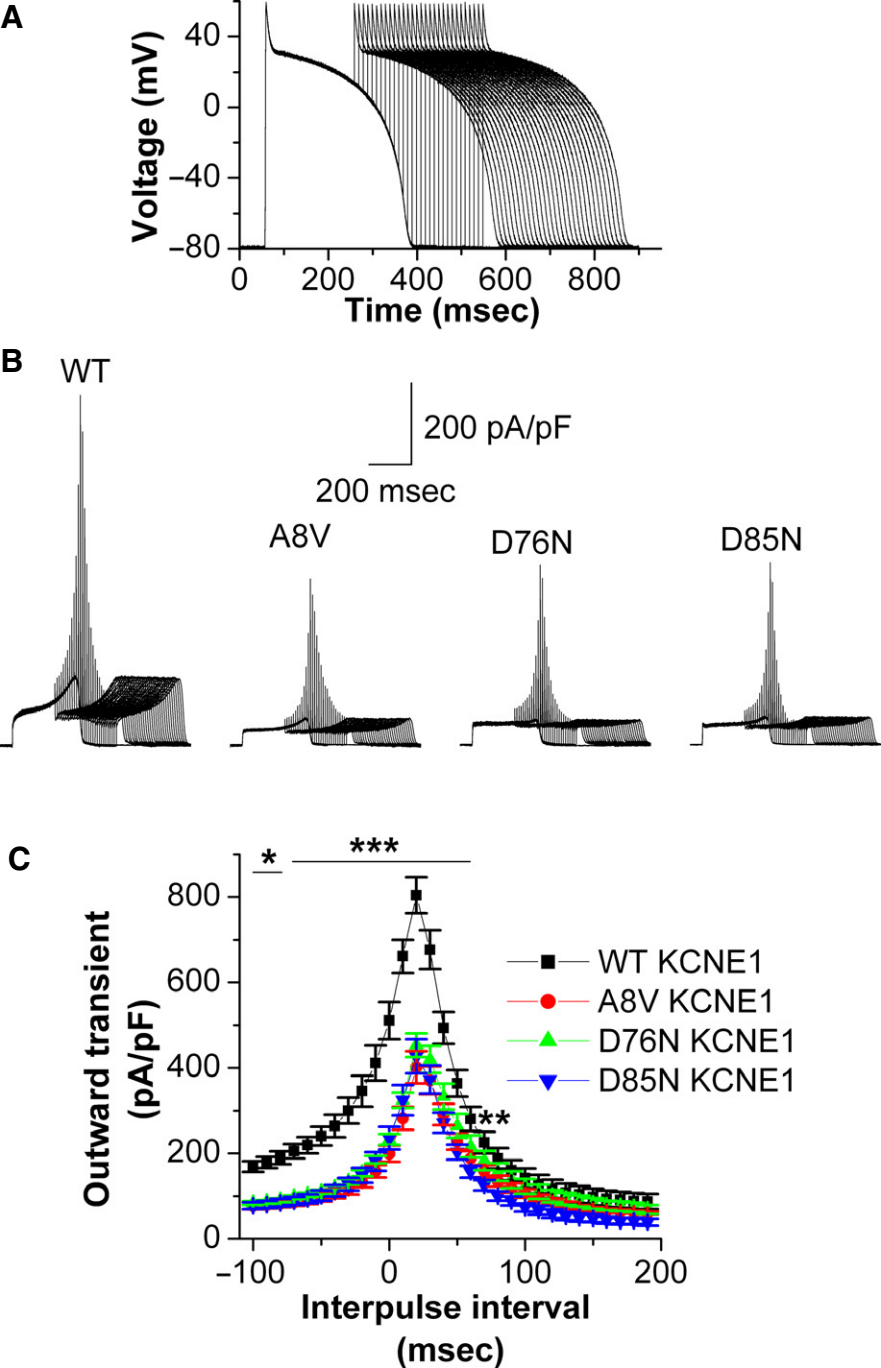
Effect of KCNE1 variants on *I*_hERG_ transients during premature action potential (AP) stimulation. (A) The protocol used to study the response of *I*_hERG_ to premature stimulation, comprised of an initial ventricular AP command followed, at varying time intervals, by a second AP waveform, superimposed on the falling phase of the initial AP and then at increasing times following repolarization. (B) Example *I*_hERG_ current traces for hERG + wild-type (WT)-KCNE1 and the three KCNE1 variants. Currents are displayed on the same scale to aid comparison. (C) Plots of the outward current transient amplitude during the paired AP waveforms, plotted against the interpulse interval. The amplitude of *I*_hERG_ transients was normalized to cell capacitance, to facilitate comparison. *n* = 6 cells for WT-KCNE1 + hERG; *n* = 7 cells for A8V-KCNE1 + hERG; *n* = 6 cells for D76N-KCNE1 + hERG; and *n* = 5 cells for D85N-KCNE1 + hERG. *P < 0.05 for A8V KCNE1, D76N KCNE1, and D85N KCNE1 compared with WT KCNE1 except for D85N KCNE1 at time point −100 msec where *P* > 0.05;**P < 0.01 for D85N KCNE1 compared with WT KCNE1; ***P < 0.001 for A8V KCNE1, D76N KCNE1, and D85N KCNE1 compared with WT KCNE1 except for D85N KCNE1 at time point 60 msec where *P* < 0.05, D76N KCNE1 at time points 50 and 60 msec where *P* > 0.05. Note that error bars for some of the points are small and are obscured by the symbols. hERG, human Ether-à-go-go-Related Gene.

The protocol for studying the response of *I*_hERG_ to premature stimuli is shown in Figure 5A. This was comprised of paired AP clamp stimuli, with the second AP applied at various intervals (both before and after complete repolarization) following the first command (cf. Lu et al. 2001; McPate et al. 2009; Du et al. 2010). Figure 5B shows families of currents elicited by this protocol for hERG + WT KCNE1 and for each of the three mutants studied. The overall pattern of response was similar for the different KCNE1 variants. In each case, the second AP command elicited rapid *I*_hERG_ transients prior to a sustained current component during the AP. The magnitude of the response first increased as the second stimulus was applied closer to 90% repolarization of the initial AP (APD_90_), with a time-dependent profile similar to that reported previously (Lu et al. 2001; McPate et al. 2009; Du et al. 2010), with responses maximal ∼20 msec after APD_90_, with the amplitude then declining. The principal difference between the *I*_hERG_ response in the presence of WT KCNE1 from that with the KCNE1 mutants was that for the three mutants the response size was markedly suppressed (with significant difference between −100 msec and +70 msec; see Fig. 5 legend for further details). This is shown clearly in Figure 5C, which shows mean data for each KCNE1 variant. Thus, the *I*_hERG_ response to premature stimuli was smaller for A8V, D76N, and D85N KCNE1 than for WT KCNE1 expression conditions.

### The effect of KCNE1 variants on the pharmacological sensitivity of *I*_hERG_

**Figure 6:**
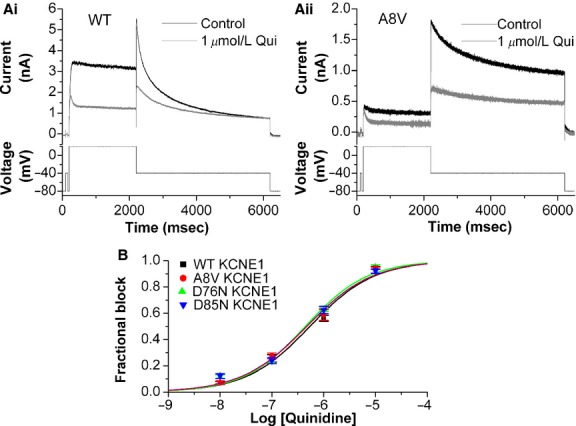
*I*_hERG_ block by quinidine for hERG with different KCNE1 variants. (A) Representative *I*_hERG_ records in the absence (black) and presence (gray) of 1 *μ*mol/L quinidine for hERG coexpressed with wild-type (WT) KCNE1 (i) and A8V KCNE1 (ii), elicited by the protocol shown in the lower panel. (B) Concentration–response plots for *I*_hERG_ inhibition by a range of quinidine concentrations for the four KCNE1 variants. The plots were fitted with a Hill equation to give IC_50_ and *h* values in the Results section. *n* ≥ 5 cells at each concentration. Note that error bars for some of the points are small and are obscured by the symbols. hERG, human Ether-à-go-go-Related Gene.

The *I*_hERG_ blocking potency of selected hERG blockers was also compared among *I*_hERG_ coexpressing WT KCNE1 and KCNE1 mutants. Figure 6A (lower traces) shows the standard protocol used, which is identical to that used in previous studies from our laboratory (McPate et al. 2008; Du et al. 2011a). We first investigated the actions of quinidine, which is an established hERG inhibitor (Lees-Miller et al. 2000; McPate et al. 2008) and has been shown to be strongly associated with acquired LQTS (Yap and Camm 2003; Hancox et al. 2008). Figure 6A-i and A-ii show representative *I*_hERG_ records in the absence and present of 1 *μ*mol/L quinidine, for WT KCNE1 and A8V KCNE1, respectively. This concentration of the drug produced similar inhibitory effects for WT KCNE1 and with each KCNE1 variant. A total of four concentrations between 10 nmol/L and 10*μ*mol/L were tested and concentration–response relations were constructed as shown in Figure 6B. Similar experiments were conducted for D76N and D85N KCNE1 and concentration–response data were plotted (Fig. 6B). These plots indicate that there was little difference in sensitivity of hERG to quinidine among the different KCNE1 variants. The derived IC_50_ values for quinidine block of *I*_hERG_ were 0.54 ± 0.13 *μ*mol/L (*h* = 0.71 ± 0.11) for WT KCNE1-hERG, 0.47 ± 0.10 *μ*mol/L (*h* = 0.69 ± 0.09) for A8V KCNE1-hERG, 0.44 ± 0.11 *μ*mol/L (*h* = 0.73 ± 0.12) for D76N KCNE1-hERG, and 0.46 ± 0.10 *μ*mol/L (*h* = 0.69 ± 0.09) for D85N KCNE1-hERG.

**Figure 7:**
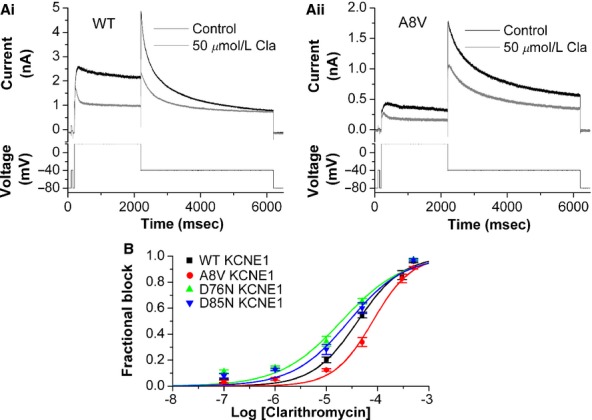
*I*_hERG_ block by clarithromycin with different KCNE1 variants. (A) Representative *I*_hERG_ records in the absence (black) and presence (gray) of 50 *μ*mol/L clarithromycin for hERG coexpressed with wild-type (WT) KCNE1 (i) and A8V KCNE1 (ii), elicited by the protocol shown in the lower panel. (B) Concentration–response plots for *I*_hERG_ inhibition by a range of clarithromycin concentrations for the four KCNE1 variants. The plots were fitted with a Hill equation to give IC_50_ and *h* values in the Results section. *n* ≥ 5 cells at each concentration. Note that error bars for some of the points are small and are obscured by the symbols. hERG, human Ether-à-go-go-Related Gene.

The *I*_hERG_ blocking effect of the macrolide antibiotic clarithromycin has been shown to differ between WT and mutant KCNE2 (Abbott et al. 1999). Therefore, we investigated whether or not its *I*_hERG_ inhibitory action may be sensitive to mutations of KCNE1. Figure 7A shows representative current records for *I*_hERG_ with WT and A8V KCNE1 before and after exposure to 50 *μ*mol/L clarithromycin. The *I*_hERG_ blocking effect of clarithromycin was lower for A8V than WT KCNE1. Figure 7B shows concentration–response data, with an IC_50_ for WT KCNE1 of 40.85 ± 4.39 *μ*mol/L, (*h* = 0.99 ± 0.09) and of 80.26 ± 9.20 *μ*mol/L (*h* = 1.15 ± 0.12) for A8V KCNE1. Thus, the A8V mutation decreased sensitivity of *I*_hERG_ to clarithromycin in comparison with that of WT KCNE1. Figure 7B also shows data for the D76N and D85N KCNE1 mutations. In contrast to the A8V mutation, both of these mutations enhanced the *I*_hERG_ inhibitory effect of clarithromycin (IC_50_ of 14.81 ± 6.76 *μ*mol/L, *h* = 0.65 ± 0.14 for D76N KCNE1 and of IC_50_ of 27.48 ± 6.77 *μ*mol/L, *h* = 0.80 ± 0.16 for D85N KCNE1).

**Figure 8:**
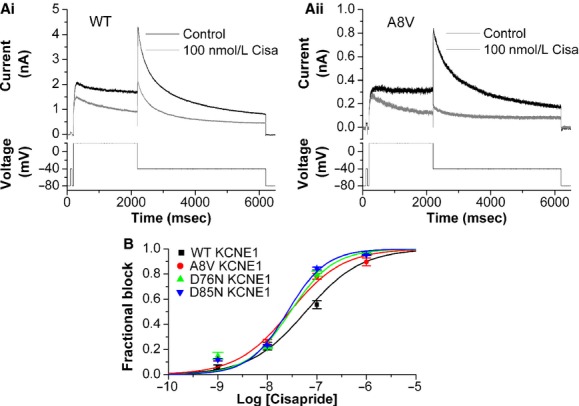
*I*_hERG_ block by cisapride with different KCNE1 variants. (A) Representative *I*_hERG_ records in the absence (black) and presence (gray) of 100 nmol/L cisapride for wild-type (WT) KCNE1 (i) and A8V KCNE1 (ii) elicited by the protocol shown in the lower panel. (B) Concentration–response plots for *I*_hERG_ inhibition by a range of cisapride concentrations for the four KCNE1 variants. The plots were fitted with a Hill equation to give IC_50_ and *h* values in the Results section. *n* ≥ 5 cells at each concentration. Note that error bars for some of the points are small and are obscured by the symbols. hERG, human Ether-à-go-go-Related Gene.

Cisapride is a gastric prokinetic drug that has been withdrawn from use due to acquired LQTS and associated torsades de pointes (TdP) arrhythmia (Henney 2000). It is a potent hERG inhibitor (Walker et al. 1999; Chen et al. 2002; Milnes et al. 2010). Figure 8A-i and A-ii show, respectively, representative *I*_hERG_ records with WT and A8V KCNE1 in the absence and presence of 100 nmol/L cisapride. The drug's action was significantly greater for A8V than WT-KCNE1 *I*_hERG_. Figure 8B shows concentration–response relations for cisapride's action for WT, A8V, D76N, and D85N KCNE1. The *I*_hERG_ blocking potency of cisapride was enhanced for the mutant KCNE1 variants compared to WT KCNE1. The IC_50_ values derived from the concentration–response relations for cisapride's action were 59.27 ± 14.86 nmol/L (*h* = 0.78 ± 0.14) for WT KCNE1-hERG, 28.13 ± 8.17 nmol/L (*h* = 0.81 ± 0.17) for A8V KCNE1-hERG, 29.08 ± 10.07 nmol/L (*h* = 1.01± 0.28) for D76N KCNE1-hERG, and 25.53 ±6.87 nmol/L (*h* = 1.12 ± 0.25) for D85N KCNE1-hERG.

## Discussion

The principal findings of this study are as follows: (1) that two KCNE1 variants associated with LQTS (A8V and D76N) and the D85N KCNE1 polymorphism decreased *I*_hERG_ amplitude compared to that with WT KCNE1, with only modest accompanying changes in *I*_hERG_ kinetics; (2) that the three KCNE1 variants studied here suppressed the *I*_hERG_ response to premature stimuli compared to the response with WT KCNE1; and (3) the sensitivity of *I*_hERG_ to pharmacological inhibition by cisapride and clarithromycin, but not quinidine, differed between variants and WT KCNE1 expression conditions.

### KCNE1 variants and *I*_hERG_ magnitude and kinetics

In the first report of the functional modulation of *I*_hERG_ by WT KCNE1, McDonald et al. (1997) found a substantial increase in macroscopic *I*_hERG_ magnitude, but with only modest alterations to WT kinetics and without changes to hERG channel unitary conductance. Furthermore, the amount of hERG protein on the cell surface was found to be unaltered by KCNE1 coexpression (McDonald et al. 1997). This led these workers to conclude that the cell membrane may normally contain pools of both active and dormant hERG channels and that KCNE1 coexpression could increase the “active fraction in a membrane pool containing active and dormant channels” (McDonald et al. 1997). Our data (Fig. 1) are in accord with those of McDonald and colleagues, in demonstrating substantially increased *I*_hERG_ density with WT KCNE1 compared to hERG alone, whereas further demonstrating that A8V, D76N, D85N KCNE1 reduced *I*_hERG_ density both compared to WT KCNE1 and to hERG alone. In respect of A8V, our AP clamp findings are in good qualitative agreement with prior conventional data for A8V-KCNE1 compared to WT KCNE1 (Ohno et al. 2007), though prior comparative data between A8V-KCNE1 + hERG and hERG alone are not available. The LQT5 D76N KCNE1 mutation has been reported to suppress *I*_hERG_ compared both to hERG alone and to hERG coexpressed with KCNE1 (Bianchi et al. 1999), whereas D85N has been reported to exert a marked dominant negative effect on *I*_hERG_ (Nishio et al. 2009; Nof et al. 2011). Our data are concordant with these findings. Our analysis of the kinetic properties of macroscopic *I*_hERG_ (Figs. 2–4) showed modest differences between hERG with WT KCNE1 and with the three KCNE1 variants studied in the voltage dependence of *I*_hERG_ availability (inactivation), while leftward shifted *I*_hERG_ activation compared to hERG expressed alone was observed for both WT KCNE1 and for the three variant KCNE1 forms studied. Thus, alterations to macroscopic *I*_hERG_ kinetics are unlikely to be primarily responsible for the differences in *I*_hERG_ density shown in Figure 1.

hERG and WT KCNE1 colocalize in membrane-processing organelles and the cell plasma membrane (Um and McDonald 2007). Although D76N KCNE1 membrane staining similar to that of WT KCNE1 has been demonstrated (Bianchi et al. 1999), hERG and KCNE1 membrane colocalization data are not yet available for the three KCNE1 variants studied. Thus, at the present time it is not known whether altered trafficking may account for or contribute to the effects of these KCNE1 variants on *I*_hERG_ density. To our knowledge, there is also a lack of information at this time regarding effects of KCNE1 variants on single-channel conductance of hERG. Further work is now warranted to determine whether either of these factors contribute to effects of KCNE1 variants on *I*_hERG_ magnitude, or whether membrane hERG protein and single-channel conductance are unaltered, and instead KCNE1 variants alter the relative proportions of active and dormant hERG channels in the membrane compared to WT KCNE1 (McDonald et al. 1997).

### Modification in the response of hERG channel to premature stimulation

hERG channels are well established to remain available early in diastole, due to rapid recovery from inactivation and comparatively slow deactivation, and can generate outward macroscopic *I*_hERG_ transients in response to depolarization; these have been proposed to exert a protective role against premature excitation (Lu et al. 2001; Vandenberg et al. 2001; McPate et al. 2009; Du et al. 2010). This protective role of *I*_hERG_ has been shown to be impaired in acidosis (Du et al. 2010) and to be altered by KCNE2 mutations associated with the LQT6 form of LQTS (Lu et al. 2003). To our knowledge, this study is the first to demonstrate a reduced response of *I*_hERG_ to premature stimulation with KCNE1 variants (Fig. 5). As *I*_hERG_ with A8V, D76N, and D85N KCNE1 exhibited modest differences in kinetics from that with WT KCNE1, the smaller *I*_hERG_ transients with these variants are likely to be a consequence of the overall reduction in *I*_hERG_ density. Our data suggest that for these three KCNE1 variants, there is a time window (between approximately ∼90 msec prior to APD_90_ and ∼60 msec post-APD_90_) during which *I*_hERG_/*I*_Kr_ would provide reduced protection from unwanted premature excitation.

### Altered *I*_hERG_ pharmacology with KCNE1 variants

To our knowledge, this is the first study to demonstrate a direct effect of KCNE1 variants on the sensitivity of hERG channels to pharmacological blockade. Questions arising from our observations are how KCNE1 exerts its modulatory effect on hERG drug sensitivity and how the responses vary between the drugs tested and between different KCNE1 variants?

The majority of hERG blocking drugs access the channel's inner cavity on channel gating and bind at a site that involves S6 aromatic amino acids and, for some drugs, interactions with other S6 and pore helical residues (Sanguinetti et al. 2005; Hancox et al. 2008). Both cisapride and quinidine exert gating-dependent *I*_hERG_ block through binding within the channel's inner cavity (Lees-Miller et al. 2000; Mitcheson et al. 2000; Sanchez-Chapula et al. 2003; Myokai et al. 2008), although quinidine block of *I*_hERG_ is less dependent on intact hERG channel inactivation than is the block with cisapride (Lees-Miller et al. 2000; McPate et al. 2008; Perrin et al. 2008). As the underlying basis of the physical interaction(s) between KCNE1 and hERG is not known at this time, it is not yet known whether and how mutations within KCNE1 N- and C-termini can influence the conformation of the hERG binding pocket. Nevertheless, there is precedence for modulation of hERG pharmacology by regulatory proteins. Thus, the membrane-associated protein KCR1 has been reported to decrease the *I*_hERG_ blocking potency of sotalol, quinidine, and dofetilide (Kupershmidt et al. 2003; Nakajima et al. 2007) and mutations to the KCNE2 protein increase the sensitivity of *I*_hERG_ to the antimicrobials sulphamethoxazole (T8A KCNE2; Nakajima et al. 2007) and clarithromycin (Q9E KCNE2; Abbott et al. 1999). The binding site on the hERG channel for clarithromycin has not yet been mapped. Although the related macrolide erythromycin interacts weakly with the canonical S6 binding residue F656 (Duncan et al. 2006), there is evidence that at least a component of clarithromycin's inhibitory action does not require hERG channel gating and so may occur to closed channels (Volberg et al. 2002). It is notable that mutations to both KCNE2 and KCNE1 alter hERG's sensitivity to clarithromycin, though in the case of KCNE1 the N-terminal A8V variant decreased rather than increased sensitivity to the drug (cf. Q9E KCNE2; Abbott et al. 1999), whereas the C-terminal D76N and D85N variants increased clarithromycin sensitivity. On the basis of our findings, further work is now needed in order to ascertain the underlying basis of the ability of KCNE1 variants to influence *I*_hERG_ pharmacology.

### Potential physiological and clinical significance

The results of this study extend the available evidence that KCNE1 and KCNE1 mutations/variants can modulate the function of hERG channels and, therefore, potentially contribute to cardiac *I*_Kr_. As these functional data have been obtained using hERG and KCNE1 overexpressed in a mammalian cell line, a question that arises is whether such interactions reflect the situation in native cardiac tissue, or are merely an artificial consequence of channel subunit overexpression in vitro? Extant evidence for physical/functional interaction between KCNE1 and hERG in native tissue is considered in the Introduction (Yang et al. 1995; Ohyama et al. 2001; Finley et al. 2002). Perhaps some of the most compelling evidence for clinically relevant *functional* modulation of hERG by KCNE1 comes from the original study reporting the A8V KCNE1 mutation, as the patient with this mutation exhibited an LQTS phenotype, whereas the A8V mutation affected KCNE1 + hERG current magnitude but not that of KCNE1 + KCNQ1 (Ohno et al. 2007). This is clearly suggestive that KCNE1 can modify or contribute to native human ventricular *I*_Kr_. Additionally, there are conflicting reports regarding the effect of D85N polymorphism on recombinant *I*_Ks_ (KCNQ + KCNE1) with both reduced current amplitude (Westenskow et al. 2004; Nishio et al. 2009) and no change in amplitude (Nielsen et al. 2007; Nof et al. 2011) reported, whereas this mutation clearly reduces *I*_hERG_ (Nishio et al. 2009; Nof et al. 2011). Thus, although KCNE1 mutations associated with QT interval prolongation may normally exert their effect through modulation of *I*_Ks_ (McCrossan and Abbott 2004; Modell and Lehmann 2006), effects mediated through *I*_Kr_ modulation can also occur. Our data support the idea that these effects can, in principle, involve a reduced contribution of *I*_Kr_ to ventricular repolarization, while showing that they may also result in a reduction in the protective role of *I*_Kr_ against premature excitation late in repolarization and early in diastole.

It is widely recognized that only a small proportion of people who receive drugs associated with acquired LQTS develop significantly prolonged QT_c_ intervals or TdP (Yap and Camm 2003; Finlayson et al. 2004; Hancox et al. 2008). Typically, patients who present with problems have additional risk factors (Viskin et al. 2003; Yap and Camm 2003; Finlayson et al. 2004; Hancox et al. 2008). KCNE1 mutations that reduce *I*_Ks_ have the potential to increase patient susceptibility to *I*_Kr_/hERG blockade through reduction in “repolarization reserve” (Roden 2008). Our findings highlight another potential means by which KCNE1 mutations/polymorphisms may increase susceptibility to drug-induced QT interval prolongation: direct modulation of the sensitivity of *I*_Kr_ to drug-block. Our data (Figs. 6–8) suggest that this may be the case for some but not all drugs and, additionally, that KCNE1 variants may have the potential both to increase and decrease hERG/*I*_Kr_ sensitivity to pharmacological inhibition by different agents. As we have investigated only three KCNE1 variants and three drugs in this study, a deal of additional future work is required in order to ascertain the extent to which other KCNE1 mutations exhibit modulatory effects similar to those reported here and which drugs are susceptible or resistant to such mutations. With that caveat, our findings in this regard are perhaps particularly noteworthy in respect of the D85N KCNE1 polymorphism. This has been linked to acquired LQTS both in individual/small group studies (Paulussen et al. 2004; Lin et al. 2012) and in larger group investigation (Kaab et al. 2012). Our data provide new evidence that, in addition to reducing *I*_Kr_ amplitude (which itself would exacerbate the effect of hERG/*I*_Kr_ blockers), D85N KCNE1 may influence acquired LQTS susceptibility to at least some drugs through a direct effect on hERG/*I*_Kr_ inhibition. Accordingly, future investigations should determine the effects of this polymorphism on *I*_hERG_ sensitivity to a wide range of drugs associated with acquired LQTS.
